# Hospital-Associated Multicenter Outbreak of Emerging Fungus *Candida auris*, Colombia, 2016

**DOI:** 10.3201/eid2507.180491

**Published:** 2019-07

**Authors:** Paige A. Armstrong, Sandra M. Rivera, Patricia Escandon, Diego H. Caceres, Nancy Chow, Matthew J. Stuckey, Jorge Díaz, Adriana Gomez, Norida Vélez, Andres Espinosa-Bode, Soraya Salcedo, Adriana Marin, Indira Berrio, Carmen Varón, Angel Guzman, Jairo E. Pérez-Franco, Julian D. Escobar, Nohora Villalobos, Juan M. Correa, Anastasia P. Litvintseva, Shawn R. Lockhart, Ryan Fagan, Tom M. Chiller, Brendan Jackson, Oscar Pacheco

**Affiliations:** Centers for Disease Control and Prevention, Atlanta, Georgia, USA (P.A. Armstrong, D.H. Caceres, N. Chow, M.J. Stuckey, A. Espinosa-Bode, A.P. Litvintseva, S.R. Lockhart, R. Fagan, T.M. Chiller, B. Jackson);; Instituto Nacional de Salud, Bogotá, Colombia (S.M. Rivera, P. Escandon, J. Díaz, A. Gomez, N. Vélez, O. Pacheco);; Clínica General del Norte, Barranquilla, Colombia (S. Salcedo, A. Marin); Clínica El Rosario, Medellín, Colombia (I. Berrio);; Corporación para Investigaciones Biológicas (CIB), Medellín (I. Berrio); Hospital General de Medellín, Medellín (I. Berrio);; Hospital Doña Pilar, Cartagena, Colombia (C. Varón, A. Guzman);; Hospital Militar Central, Bogotá (J.E. Pérez-Franco, J.D. Escobar);; Clínica los Nogales, Bogotá (N. Villalobos, J.M. Correa)

**Keywords:** fungi, candidemia, antimicrobial resistance, healthcare-associated, Colombia, nosocomial infections, *Candida auris*

## Abstract

*Candida auris* is an emerging multidrug-resistant fungus that causes hospital-associated outbreaks of invasive infections with high death rates. During 2015–2016, health authorities in Colombia detected an outbreak of *C. auris*. We conducted an investigation to characterize the epidemiology, transmission mechanisms, and reservoirs of this organism. We investigated 4 hospitals with confirmed cases of *C. auris* candidemia in 3 cities in Colombia. We abstracted medical records and collected swabs from contemporaneously hospitalized patients to assess for skin colonization. We identified 40 cases; median patient age was 23 years (IQR 4 months–56 years). Twelve (30%) patients were <1 year of age, and 24 (60%) were male. The 30-day mortality was 43%. Cases clustered in time and location; axilla and groin were the most commonly colonized sites. Temporal and spatial clustering of cases and skin colonization suggest person-to-person transmission of *C. auris*. These cases highlight the importance of adherence to infection control recommendations.

## Abstract 2

Adherence to infection control recommendations is critical to prevent spread of this novel yeast.

*Candida auris* is an emerging multidrug-resistant fungus that has been implicated in recent hospital-associated outbreaks of invasive infections with high death rates (*1*). *C. auris* from an external ear isolate in Japan was described in 2009; the fungus has since been identified as the cause of outbreaks in other countries, with rapid spread globally (*2*–*5*). Of particular concern, *C. auris* isolates have demonstrated resistance to multiple classes of antifungal drugs (*2*,*6*). In addition, whereas *Candida* is often considered a commensal organism that most commonly colonizes the gastrointestinal tract, the potential for person-to-person spread of *C. auris* has raised concern for widespread outbreaks (*7*,*8*).

Accurate identification of *C. auris* is challenging because phenotypic methods do not correctly identify this species. It is most commonly misidentified as *C. haemulonii*, especially when using the VITEK 2 system (bioMérieux, https://www.biomerieux-diagnostics.com), but is also misidentified as *C. famata*, *C. sake*, *Rhodotorula glutinis*, and other *Candida* species (*9*). Specialized techniques, such as matrix-assisted laser desorption/ionization-time of flight (MALDI-TOF) mass spectrometry and DNA sequencing, are needed for reliable identification.

During January 2015–September 2016, the Colombian Instituto Nacional de Salud (INS) and the Secretarias de Salud (Ministries of Health) of Barranquilla, Bogotá, and Cartagena, Colombia, identified an increasing number of reported bloodstream infections with *C. haemulonii*, an otherwise rare yeast isolated in only a few invasive human infections (*10*). In May 2016, a total of 27 isolates initially identified as *C. haemulonii* by phenotypic identification methods were sent to the US Centers for Disease Control and Prevention (CDC), where 24 were confirmed to be *C. auris* by MALDI-TOF mass spectrometry (Microflex; Bruker Daltonics, https://www.bruker.com) and DNA sequencing. Through case findings and review of national surveillance data, an additional 16 cases of *C. haemulonii* reported to INS were identified as *C. auris* by MALDI-TOF mass spectrometry and confirmed at CDC with sequencing. The cases were reported from 4 large referral hospitals in Colombia. Two of these hospitals were located along the northern coast: 1 in Barranquilla, which serves both pediatric and adult patients, and the other a specialized pediatric facility in Cartagena. The other 2 hospitals were located in Bogotá and serve adult patients. No transfers took place between the involved facilities, and all follow national guidelines for infection control practices (*11*). These findings prompted an investigation to further characterize the epidemiology, transmission mechanisms, and environmental reservoirs of this organism to guide prevention measures.

## Methods

In September 2016, a team consisting of clinicians, epidemiologists, and microbiologists from INS, CDC, and the Ministries of Health of Barranquilla, Bogotá, and Cartagena conducted an outbreak investigation in 1 acute-care hospital serving adult and pediatric patients in Barranquilla, 2 adult hospitals in Bogotá, and 1 pediatric hospital in Cartagena. These facilities all had confirmed cases of *C. auris* candidemia after January 2015.

### Descriptive Epidemiology

We defined a case as *C. auris* confirmed by MALDI-TOF mass spectrometry isolated from a patient’s blood during January 2015–September 2016. We abstracted medical records using a standardized case report form to collect data on concurrent conditions, location and duration of hospital stay, administration of antimicrobial drugs, exposure to invasive procedures, use of indwelling catheters, and outcome. We also interviewed infection control personnel, healthcare workers, and key informants at each site to trace exposures and locations of patients throughout their hospital stays.

### Sampling and Laboratory Analysis

To better understand the role of colonization and transmission, we obtained samples from the 7 patients who were hospitalized at the time of the investigation with *C. auris* isolated or suspected in a specimen. We used premoistened swabs (Fisherfinest Transport Swabs, https://www.fishersci.com) to sample the following 10 body sites: bilateral nares, ears, axillae, and groin; oral cavity; and rectum. These locations were determined to be high-yield sites for colonization, and sampling was intended to provide initial insight into the utility of each. We collected fecal and urine specimens when possible.

Specimens were processed at the INS Microbiology Laboratory using protocols developed by CDC’s Mycotic Diseases Branch (National Center for Emerging and Zoonotic Infectious Diseases, Division of Foodborne, Waterborne, and Environmental Diseases). In brief, the swabs were cultured in Sabouraud dextrose enrichment broth with high salt content (10% wt/vol NaCl) at elevated temperature (40°C), shaken (250 rpm) for up to 7 days, and plated onto Sabouraud dextrose agar (*12*). Yeast isolates recovered from the selective agar were initially screened using CHROMagar Candida (Becton Dickinson, http://www.bd.com); white and pink colonies were typed using the Microflex database version MBT 6903 MSP Library (no. 1829023) using CDC MicrobeNet’s supplemental MALDI database library.

Antifungal susceptibility testing was performed at CDC by broth microdilution using custom-made frozen panels; susceptibility testing for amphotericin B was performed using Etest (bioMérieux). We used the following breakpoints, based on published data: fluconazole >32 μg/mL, amphotericin B >2 μg/mL, caspofungin >4 μg/mL, and anidulafungin >2 μg/mL (*13*).

### Statistical Analysis

We calculated medians and ranges for continuous variables and frequencies and percentages for categorical variables. We used 30-day mortality as outcome of interest for analysis. To compare characteristics between patients who died and those who survived, we applied a *t*-test (equal or unequal variance as appropriate) for continuous variables and a χ^2^ test for categorical variables. For categorical variables having cell sizes <5, we used the Fisher exact test. Sample size did not allow for multivariate analysis. When comparing medians, we used Wilcoxon rank, as distributions were not normal. We performed all data analyses in SAS version 9.3 (https://www.sas.com) and considered a p value <0.05 significant.

CDC and INS determined that this was an emergency public health investigation. Therefore, it did not meet the criteria for research. 

## Results

We identified 40 cases of *C. auris* candidemia occurring during January 2015–September 2016. In 3 hospitals, cases clustered in May–July 2016, whereas in the fourth hospital, cases occurred throughout the 21-month period (Figure). The median patient age was 23 years (interquartile range [IQR] 4 months–56 years); 12 patients (30%) were <1 year of age. Most patients (24; 60%) were male. Thirty-five (88%) patients had a documented concurrent condition before hospitalization; the most commonly reported conditions were hemodialysis for renal failure (23%); diabetes (18%); and immunocompromising conditions (16%), including cancer and transplant. 

**Figure:**
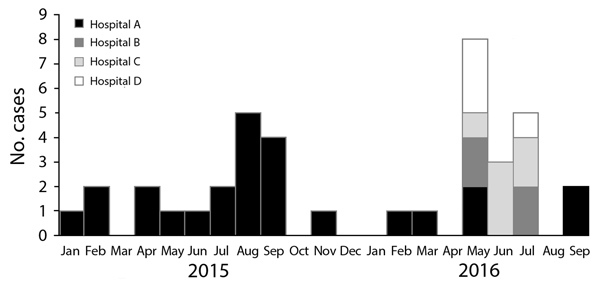
Epidemic curve for cases of *Candida auris* candidemia in Colombia, by hospital, January 2015–September 2016.

More than half (58%) of patients died while hospitalized; the overall 30-day mortality was 43%. The median length of admission was 46 days (IQR 34–69 days). For the 38 patients with known intensive care unit (ICU) admission, median length of ICU stay was 36 days (IQR 25–58 days). All case-patients were exposed to an invasive procedure. Central venous catheter (CVC) placement was present in all 40 patients (100%); other common procedures were intubation (35; 97%), surgical procedures (28; 70%), and hemodialysis (15; 38%) (Table 1). 

**Table 1:** Characteristics of 40 patients with *Candida auris* bloodstream infection, Colombia, 2015–2016*

Characteristic	Value	Data missing
Median age (IQR)	23 y (4 mo–56 y)	0
Infant up to 1 y	12 (30)	0
Child 1–18 y	8 (20)	0
Adult 18–65 y	14 (35)	0
Adult >65 y	6 (15)	0
Sex		
M	24 (60)	0
F	16 (40)	0
Concurrent conditions	35 (88)	0
Chronic renal disease	9 (23)	0
Hemodialysis dependent	9 (23)	0
Diabetes	7 (18)	2
Immunosuppressive condition	6 (16)	3
Cancer	4 (11)	3
Solid tumor	2 (5)	4
Hematologic malignancy	2 (5)	4
Transplant	2 (5)	4
Neuromuscular condition	1 (3)	0
Outcome at 30 d		
Deceased	17 (43)	0
Alive	21 (53)	0
Hospitalized	2 (5)	0
In-hospital deaths	23 (58)	6
Admitted to hospital	40 (100)	0
Median inpatient stay, d (IQR)	46 (34–69)	3
Admitted to ICU	38 (100)	2
Median ICU stay, d (IQR)	36 (25–58)	10
Transferred from another facility	16 (40)	0
Previously hospitalized in the 90 d before admission		
Yes	11 (28)	1
No	23 (59)	1
Unknown	5 (13)	1
Previous exposure to antifungal drugs		
No	22 (67)	7
Unknown	11 (33)	7
Treatments and procedures		
CVC	40 (100)	0
Vasopressors	31 (82)	2
Respiratory support	36 (100)	4
Intubation	35 (97)	4
Bilevel positive airway pressure	1 (3)	4
Surgical procedure	28 (70)	0
Total parenteral nutrition	19 (50)	2
Corticosteroids	18 (47)	2
Hemodialysis	15 (38)	1
Bronchoscopy	7 (19)	4
Chemotherapy	4 (11)	3
Median time from admission to *C. auris* positive culture, d (IQR)	22 (18–31)	0
Median time from CVC to *C. auris* positive culture, d (IQR)	12 (5–21)	1
Treatment for *C. auris* bloodstream infection		0
Fluconazole	16 (42)	
Caspofungin	13 (34)	
Amphotericin B	8 (21)	
Voriconazole	1 (3)	
No antifungal	2 (5)	
>1 antifungal	19 (48)	
No. antibacterial drugs administered, median (range)	3.5 (1–6)	

*Values are no. (%) patients except as indicated. CVC, central venous catheter; ICU, intensive care unit; IQR, interquartile range.

Median time from admission to having a blood culture positive for *C. auris* was 22 days (IQR 18–31 days). The median time from placement of CVC to positive blood culture was 12 days (IQR 5–21 days). The most common treatments received for *C. auris* candidemia were fluconazole (16; 43%) and caspofungin (13; 34%). Nineteen patients (45%) received >1 antifungal drug; the median number of antimicrobial drugs administered to patients was 3.5 (range 1–6) (Table 1). The most frequent laboratory abnormalities were mild leukocytosis (median leukocyte count 12.7 × 10^9^ cells/L) and anemia (median hemoglobin 9.8 g/dL).

The median age of the 17 patients who died within 30 days of positive culture was 36 years (IQR 6 months–65 years). Neither age group (p = 0.18), sex (p = 0.9), nor preexisting concurrent condition (p = 0.30) were associated with 30-day mortality. Diabetes was associated with 30-day mortality (odds ratio [OR] 12, 95% CI 1.28–112.42; p = 0.03). The median length of hospital stay was longer for surviving case-patients (63 days, IQR 45–95 days) than for those who died (36 days, IQR 29–45 days; p<0.01). The median length of ICU admission was 32 days (IQR 27–44 days) for case-patients who died and 51 days (IQR 14–76 days) for those who survived (p = 0.3). No procedures were found to be associated with 30-day mortality. The median time from admission to positive blood culture was 22 days (IQR 21–37 days) for the patients who died and 21 days (IQR 12–28 days) in those who survived (p = 0.23). The median time from positive blood culture to death in those who died was 7.5 days (IQR 6–17 days). We were unable to ascertain whether death was attributable to *C. auris* fungemia.

Among the 12 cases in infants, median age was 34 days (IQR 17–107 days) and 9 (75%) were male; 6 (50%) died in the hospital, and 5 (42%) died within 30 days of *C. auris* culture. Five (50%) were preterm (<37 weeks’ gestation), and 6 (50%) had congenital heart disease (Table 2). Four (80%) of 5 premature infants survived.

**Table 2:** Characteristics of 12 infants <1 y of age with *Candida auris* bloodstream infection, Colombia, 2015–2016*

Characteristic	Value	Data missing
Median age, d (IQR)	34 (17–107)	0
Sex		
M	9 (75)	0
F	3 (25)	0
30-day mortality	5 (42)	0
In-hospital mortality		
Died	6 (50)	0
Alive	4 (33)	0
Still hospitalized, 2016 Sep 30	2 (17)	0
Concurrent conditions	12 (100)	0
Prematurity	5 (50)	2
Median gestational age at birth, wk (IQR)	36 (29–39)	2
Congenital heart disease	6 (50)	0
Surgical procedure	9 (75)	0
Treatments and procedures		
Central venous catheter	12 (100)	0
Intubation	11 (100)	1
Total parenteral nutrition	11 (92)	0
Feeding tube	5 (71)	5
Corticosteroids	4 (33)	0
Hemodialysis	1 (8)	0

*Values are no. (%) patients except as indicated. CVC, central venous catheter; IQR, interquartile range.

We performed antifungal susceptibility testing on 34 available clinical isolates from 34 unique case patients at 3 hospitals. Six (18%) isolates were resistant to fluconazole, 10 (29%) to amphotericin B, and none to the echinocandin class (anidulafungin, caspofungin). Ten (50%) patients who died had been treated with fluconazole, 9 (43%) received caspofungin, 1 received amphotericin B, and 1 received voriconazole; 2 patients who died did not receive an antifungal drug. Of the 6 case-patients with isolates resistant to fluconazole, 4 (67%) died by 30 days. Of the 10 case-patients with isolates resistant to amphotericin B, 4 (40%) died by 30 days. Resistance to fluconazole was not associated with outcome (p = 0.21), nor was resistance to amphotericin B (p = 0.85) (Table 3). Fluconazole-resistant isolates were seen at all 3 hospitals and amphotericin B-resistant isolates at 2 hospitals, in Barranquilla and Cartagena (northern region of Colombia).

**Table 3:** Characteristics of patients who died at 30 d compared with those who survived *Candida auris* bloodstream infection, Colombia, 2015–2016*

Characteristic	Total	Died	Survived	Crude odds ratio (95% CI)	p value†
All	40 (100)	17 (43)	23 (58)	NA	NA
Age, y					0.18
<1	12 (30)	5 (29)	7 (30)	0.95 (0.24–3.75)	0.94
1–17	8 (20)	2 (12)	6 (26)	0.24 (0.04–1.45)	0.12
18–65	14 35)	5 (29)	9 (39)	0.65 (0.17–2.47)	0.53
>65	6 (15)	5 (29)	1 (4)	9.17 (0.96–87.79)	0.05
Sex				0.92 (0.26–3.30)	0.9
M	24 (60)	10 (59)	14 (61)		
F	16 (40)	7 (41)	9 (39)		
Concurrent conditions	35 (88)	16 (94)	19 (83)	3.37 (0.34–33.26)	0.30
Chronic renal disease	9 (23)	3 (18)	6 (26)	0.61 (0.13–2.88)	0.53
Hemodialysis dependent	9 (23)	3 (18)	6 (26)	0.61 (0.13–2.88)	0.53
Diabetes	7 (18)	6 (35)	1 (4)	12 (1.28–112.42)	0.03
Immunosuppressive condition	6 (16)	4 (24)	2 (9)	3.23 (0.52–20.20)	0.21
Prematurity, n = 12	5 (42)	1 (6)	4 (17)	0.30 (0.03–2.93)	0.30
Median length of hospital stay, d (IQR)	46 (34–69)	36 (29–45)	63 (45–95)	NA	<0.01
Median length of ICU stay, d (IQR)	36 (25–58)	32 (27–44)	51 (14–76)	NA	0.3
Central venous catheter	40 (100)	17 (100)	23(100)	NA	NA
Respiratory support	36 (90)	17 (100)	19 (83)	NA	0.12
Vasopressors	31 (82)	16 (94)	15 (65)	8.53 (0.95–76.63)	0.06
Surgical procedure	28 (70)	13 (76)	15 (65)	1.73 (0.43–7.11)	0.45
Total parenteral nutrition	19 (50)	7 (41)	12 (52)	0.64 (0.18–2.28)	0.49
Corticosteroids	18 (47)	8 (47)	10 (43)	1.16 (0.33–4.07)	0.82
Hemodialysis	15 (38)	8 (47)	7 (30)	2.03 (0.55–7.47)	0.29
Chemotherapy	4 (11)	2 (12)	2 (9)	1.4 (0.18–11.08)	0.74
Median time from admission to *C. auris* positive culture, d (IQR)	22 (18–31)	22 (21–37)	21 (12–28)	NA	0.23
Isolates resistant to fluconazole, n = 34	6 (18)	4 (24)	2 (9)	3.23 (0.52–20.2)	0.21
Isolates resistant to amphotericin B, n = 34	10 (29)	4 (24)	6 (26)	0.87 (0.20–3.74)	0.85

*Values are no. (%) patients except as indicated. ICU, intensive care unit; IQR, interquartile ratio; NA, not applicable. †Fisher exact test, χ^2^, *t*-test, or Wilcoxon rank as appropriate.

### Patient Sampling

We collected skin and rectal swab specimens from 7 patients admitted at the time of the investigation: 3 patients with *C. auris* bloodstream infection, 2 with *C. auris* cultured from a body site other than blood, and 2 without a clinical *C. auris* culture (but who at the time had a clinical culture growing yeast suspected to be *C. auris*). Five patients yielded positive samples, 4 of whom had a clinical culture with *C. auris*. One patient with a bloodstream infection had no positive samples. Samples were collected 3 weeks to 3 months after positive clinical culture (Table 4). The case isolates showed a high degree of relatedness on whole-genome sequencing within hospitals and regional clustering, supporting in-hospital and person-to-person transmission (*14*).

**Table 4:** Patient sampling in suspected or known cases of *Candida auris* fungemia, Colombia, 2016*

Patient no.	Primary specimen type positive	No. body sites sampled for colonization	No. (%) body sites positive for *C. auris*
1	Blood	11	1 (9%): rectum
2	Blood	10	0
3	Blood	11	7 (64%): ears, axilla, left nostril, rectum, fecal material
4	Urine	10	1 (10%): left groin
5	Sputum	11	1 (9%): right groin
6	None	10	2 (20%): axillae

*Sampling from a seventh patient, who had an unidentified yeast growing in blood culture at the time of testing, did not yield *C. auris*.

We identified spatial and temporal clustering of cases in 1 operating room and multiple intensive care units at all 4 sites. Hospital A (Figure) experienced the largest number of cases over the longest period. Few case-patients shared the same room, and cases occurred throughout different floors and units. For the remaining hospitals, all cases occurred within a 3-month period. At hospital B (Figure), all 4 cases were exposed to the same operating room over a 3-month period. In hospitals C and D, patients also overlapped in time and ICUs (Figure).

## Discussion

Cases of *C. auris* bloodstream infection in Colombia were associated with nearly 60% all-cause, in-hospital deaths and a 43% 30-day mortality, which is higher than that reported in the United States. Cases occurred primarily in patients in ICUs who had central venous catheters and other invasive devices, continuing to support the findings that these cases occur in patients with long stays involving multiple procedures (*13*). In contrast to the United States, where most cases have occurred in older adults, nearly one third of cases in Colombia were in infants, and the median patient age was 23 years (*15*). Although cases are occurring in vulnerable extremes of age populations, our findings suggest that no age group is unaffected and that any given hospital could be affected by an outbreak.

Clustering of patients in time and space, the findings of skin colonization, and the highly clonal nature of the isolates all strongly support person-to-person transmission occurring in the healthcare setting. Although *Candida* is often considered a commensal organism that most commonly colonizes the gastrointestinal tract, *C. auris* behaves more similarly to *C. parapsilosis* in its propensity to colonize the skin, which provides an opportunity for person-to-person spread. Outbreaks of *C. parapsilosis* have occurred in both adult and neonatal ICUs (*16*,*17*). In our investigation, 2 NICUs were involved; in at least 1 NICU, healthcare workers shared time between patients, providing an opportunity for transmission. The propensity of *C. auris* to colonize the skin may also explain the strong association with intubation, catheters, and feeding tubes in infants. The ability of *C. auris* to form biofilms may further enhance its ability to migrate into the bloodstream when provided a conduit through the skin (*18*). In this investigation, central lines were in place for a mean of 12 days at the time of positive culture. The tendency for skin colonization, biofilm production, and ability to cause invasive infection further emphasizes the need for diligent central line care. 

The high death rate associated with *C. auris* infections, coupled with potential for nosocomial transmission, including among high-risk neonates, underscores the importance of infection control to prevent its spread. Current recommendations focus on early notification, hand hygiene, disinfection with a US Environmental Protection Agency–registered hospital-grade disinfectant effective against *Clostridium difficile* spores, and use of standard and contact precautions (*19*).

The characteristics of patients with *C. auris* candidemia were similar, but not identical, to those reported for candidemia caused by other species. Patients with *C. auris* bloodstream infection shared exposures such as ICU stay, central venous catheters, and surgical procedures (*7*). Whereas immunosuppressive conditions, including solid organ tumors, hematologic malignancy, and transplants are known risk factors for candidemia, they were uncommon in patients with *C. auris* candidemia (*18*). The reason for this may be that whereas other *Candida* species invade when there is a shift in the physiology of the host, *C. auris* is not a commensal, but rather a new exposure. Thirty-day mortality rates for *C. auris* candidemia in Colombia appear to be slightly higher (43%) than those seen for candidemia from other species, although the small sample size in our investigation limits the comparison (*20*). Half the infants in our cohort died, although age was not significantly associated with death. Furthermore, we compared characteristics between case-patients who survived and those who died and found few to be associated with outcome. Preterm birth was more common in those who survived, and only diabetes was associated with 30-day mortality. The high death rate is likely multifactorial but is most clearly related to long hospital stays, multiple invasive procedures, and lack of clinician awareness of resistance profiles.

In September 2016, a national laboratory alert was released in Colombia to increase reporting and surveillance catchment, as well as to enhance awareness of *C. auris* and the other species with which it was most commonly confused (*21*). According to the national guidelines for surveillance of healthcare-associated infections, aerobic and anaerobic blood cultures should be performed on any patient with signs of bloodstream infection (*11*). However, the capacity to perform blood cultures may not be available in all facilities; thus, it is possible that cases may be missed. The alert coincided with the initiation of our investigation and thus did not contribute heavily to our case counts, but it did increase reporting nationally.

Directed treatment is key to successful outcomes in fungemia. Resistance is another noteworthy feature of *C. auris*. Among *C. auris* isolates collected from 4 world regions, nearly all (93%) were resistant to fluconazole, and 41% were resistant to >2 classes of antifungal drugs (*13*). In our investigation, fluconazole was the most common antifungal drug administered, and treatment failure may have played a role in death rates. Of the isolates in this investigation, only about one fifth were resistant to fluconazole, but nearly one third were resistant to amphotericin B. The low proportion resistant to fluconazole was surprising; medical practices surrounding administration likely play a role. Forty-three percent of patients received fluconazole around the time of blood culture yielding *C. auris*, and of these, 57% died. The mortality rate was even higher (83%) among the patients with isolates resistant to fluconazole. Most *C. auris* isolates have been susceptible to echinocandin drugs, which remain the recommended first-line treatment for *C. auris* candidemia (*9*,*13*,*22*). However, the often high cost of these drugs can be an obstacle to their use in resource-limited settings.

Because of the retrospective nature of this investigation, data on all variables were not available for each case-patient. Data on previous exposure to antifungal drugs and laboratory values were the most commonly missing. These limitations, along with the small sample size, may have prevented the detection of associations between risk factors and outcome.

The findings of our investigation highlight the necessity of adherence to infection control recommendations, especially aspects of careful central line care and maintenance, hand hygiene, proper disinfection of medical equipment, and use of standard and contact precautions (https://www.cdc.gov/fungal/diseases/candidiasis/recommendations.html). *C. auris* remains an emerging pathogen with the potential for high levels of resistance to a limited body of antifungal drugs. Its propensity to colonize skin provides a means for person-to-person transmission and elevates the concern for healthcare-associated outbreaks. Further understanding of its comportment and attention to infection control and prevention recommendations are paramount to prevent further spread.
